# 

**Published:** 1915-05-22

**Authors:** 


### Medical Appointments.

Miss L. M. Smith Clark, M.B., Ch.B. Edin., has been appointed by the Metropolitan Asylums Board an assistant medical officer in the infectious hospital service.

Miss Frances M. Huxley, B.Sc., M.D., has been appointed registrar, and reappointed house surgeon to the Samaritan Free Hospital for Women, Marylebone Road, for a period of six months; Mrs. Margaret Brady, M.B., B.S., has been reappointed anæsthetist for six months; and Miss Mary Alice Blair, M.D., B.S., has been appointed anæsthetist for six months.

Dr. William J. P. Lillis has been appointed by the Bermondsey Board of Guardians *locum tenens* for Dr. F. S. Crean, assistant medical officer at their infirmary, at a salary of £7 7s. a week, with the usual residential allowances. The visiting committee reported that they found it quite impossible to obtain the services of a medical practitioner below that salary, which was the present minimum rate of pay for *locum tenens*.

Dr. Richard Hamilton Townend, M.D. Lond., M.R.C.S. Eng., L.R.C.P. Lond., of Adelaide Road, Brockley, has been appointed by the Bermondsey Board of Guardians temporary medical officer at the Ladywell Institution at a salary of £5 5s. weekly during the absence of Dr. J. T. Macnamara, who has accepted service in the Royal Army Medical Corps. The board have decided that the supervision, control, and charge of the medical arrangements at the institution shall be entrusted to Dr. E. S. Bell, the medical superintendent of their infirmary. At first the board was desirous that Dr. Bell should with his staff undertake the whole of the medical work at the institution, but after visiting the institution he came to the conclusion that he could only do this at the expense and neglect of his work in the infirmary, where he was severely handicapped by the absence of Dr. Eccles and Dr. Randle, and their replacement by substitutes. He was prepared to accept nominal responsibility for the medical work, with the assistance of Dr. Townend, and he was of opinion that by degrees it could be placed on a better footing, and more satisfactory arrangements made for the location, classification, and transfer of patients generally between the various institutions of the Guardians.

### Order of St. John.

It is notified in the *London Gazette* that the King has sanctioned the following promotions in and appointments to the Order of the Hospital of St. John of Jerusalem in England:

As Honorary Knight of Justice.—The King of the Belgians.

As Honorary Lady of Justice.—The Queen of the Belgians.

As Lady of Justice (from Lady of Grace).—Adeline Duchess of Bedford.

As Knights of Grace.—The Hon. Sir George Edward Knox; Lieut.-Colonel Sir Arthur Henry McMahon, G.C.V.O., K.C.I.E., C.S.I.; Sir Henry Parsall Burt, K.C.I.E.; Lord Stanmore; Sir David Yule Yule; Sir Maurice William Ernest de Bunsen, P.C., G.C.M.G., G.C.V.O., C.B.; Evelyn Cecil, M.P., Sir Louis William Dane, G.C.I.E., C.S.I.; and Sir Charles Cheers Wakefield.

As Ladies of Grace.—Anne Elfleda, Mrs. Waynman Dixon; Helen Sophia, Lady Sloggett; Amy Sarah, Miss Hughes (from Honorary Serving Sister); Mary Venetia, Mrs. Arthur James; Margaret Carlotta, Mrs. F. W. Westmacott.

As Esquires.—Major John Bernard Arbuthnot, M.V.O., Stewart Gordon.

Rates of Subscription : Twelve months, 6/6 ; Six months, 4/- ; Three months, 2/-, post free to any address in the United Kingdom. Abroad, Twelve months, 8/8; Six months, 5/-; Three months, 2/6, post free.—Address The Subscription Dept., "The Hospital," The Hospital Building, 28/29 Southampton Street, Strand, London, W.C.

